# A comparative study of teriflunomide and dimethyl fumarate within the Swedish MS Registry

**DOI:** 10.1177/13524585211019649

**Published:** 2021-06-03

**Authors:** Jan Hillert, Jon A Tsai, Mona Nouhi, Anna Glaser, Tim Spelman

**Affiliations:** Department of Clinical Neuroscience, Karolinska Institute, Stockholm, Sweden; Sanofi, Stockholm, Sweden; Sanofi, Stockholm, Sweden; Department of Clinical Neuroscience, Karolinska Institute, Stockholm, Sweden; Department of Clinical Neuroscience, Karolinska Institute, Stockholm, Sweden

**Keywords:** Dimethyl fumarate, teriflunomide, treatment response, multiple sclerosis

## Abstract

**Background:**

Teriflunomide and dimethyl fumarate (DMF) are first-line disease-modifying treatments for multiple sclerosis with similar labels that are used in comparable populations.

**Objectives:**

The objective of this study was to compare the effectiveness and persistence of teriflunomide and DMF in a Swedish real-world setting.

**Methods:**

All relapsing-remitting multiple sclerosis (RRMS) patients in the Swedish MS registry initiating teriflunomide or DMF were included in the analysis. The primary endpoint was treatment persistence. Propensity score matching was used to adjust comparisons for baseline confounders.

**Results:**

A total of 353 teriflunomide patients were successfully matched to 353 DMF. There was no difference in the rate of overall treatment discontinuation by treatment group across the entire observation period (hazard ratio (HR) = 1.12; 95% confidence interval (CI) = 0.91–1.39; *p* = 0.277; reference = teriflunomide). Annualised relapse rate (ARR) was comparable (*p* = 0.237) between DMF (0.07; 95% CI = 0.05–0.10) and teriflunomide (0.09; 95% CI = 0.07–0.12). There was no difference in time to first on-treatment relapse (HR = 0.78; 95% CI = 0.50–1.21), disability progression (HR = 0.55; 95% CI = 0.27–1.12) or confirmed improvement (HR = 1.17; 95% CI = 0.57–2.36).

**Conclusion:**

This population-based real-world study reports similarities in treatment persistence, clinical effectiveness and quality of life outcomes between teriflunomide and dimethyl fumarate.

## Introduction

Multiple sclerosis is a chronic inflammatory condition of the central nervous system affecting more than 2 million people worldwide.^1^ The most common phenotype of MS is the relapsing form (RMS), for which there are approximately 20 currently approved treatments. Within first-line therapy, the oral medications teriflunomide and dimethyl fumarate (DMF), introduced in 2013–2014, have largely replaced platform injectable therapies. Teriflunomide has previously been investigated in five clinical trials; two placebo-controlled in RMS, one placebo-controlled trial in clinically isolated syndrome (CIS) and a single direct comparison against interferon-beta in RMS.^2–5^ The clinical development programme for DMF involved two RMS placebo-controlled trials.^6,7^ Both teriflunomide and DMF were associated with significant reductions in relapse rates relative to placebo. In relative terms, the reduction in relapse rate was larger for DMF, 53% in DMF and 44% placebo, compared to teriflunomide (32% and 36%). The absolute reduction of annualised relapse rate (ARR) was similar for these two treatments, 0.19 and 0.18 for DMF and 0.17 and 0.18 for teriflunomide (*p* < 0.001 in all studies). Teriflunomide was associated with a significant reduction in confirmed disability worsening relative to placebo in both RMS trials (20% teriflunomide vs 27% placebo (*p* = 0.028) and 16% vs 20% (*p* = 0.044), respectively). By comparison, DMF was associated with a similar reduction in the rate of confirmed disability worsening in just one of the two placebo-controlled trials (16% DMF vs 27% placebo (*p* = 0.005)). No such difference was observed in the other placebo-controlled trial, 13% versus 17% (*p* = 0.25).

Real-world studies are important for understanding the performance of new disease-modifying treatments following their approval. Teriflunomide and DMF have similar European Medicines Agency (EMA) and Food and Drug Administration (FDA) labels and are therefore suitable for comparison in the first-line setting. These two oral therapies have been previously compared in a small number of cohorts employing a range of different study designs and methodologies. An Italian multi-centre study from 2018 found a similar effect of these two treatments with regard to both relapse rates and no evidence of disease activity (NEDA).^8^ A more recent Italian comparison of 1445 patients treated with either DMF or teriflunomide reported higher rates relapse-free survival in patients on DMF after 38 months of treatment.^9^ A 2019 study from the French Observatoire Français de la Sclérose en Plaques (OFSEP) registry found less magnetic resonance imaging (MRI) activity in the DMF group relative to teriflunomide, although no difference was observed in either relapse rates or disability worsening.^10^ Another study from the Danish Multiple Sclerosis Registry from 2019, which excluded patients who had been treated for MS more than 8 years, demonstrated less relapses with DMF compared to teriflunomide, but no difference in terms of disability outcomes.^11^

The objective of this study was to compare real-world effectiveness and treatment persistence in a propensity score matched cohort of teriflunomide and DMF-treated patients from the Swedish MS Registry. The registry covers more than 80% of the MS population in Sweden on disease-modifying treatments.^12^ A comparison of quality of life (QoL) outcomes is also presented in a subgroup of patients.

## Materials and methods

### Data

All data used in the analysis were sourced from the Swedish Multiple Sclerosis registry (SMSreg). Data for the analysis were extracted from the registry on 25 November 2019. The registry was established in 2000 to capture and collate clinical data on multiple sclerosis patients over time.^12^ While participation by neurologists in the registry operates on an opt-in basis, SMSreg is currently used in all 60 MS care units across Sweden capturing approximately 80% of the prevalent Swedish MS population. A minimum dataset of mandatory variables is required for data upload and includes patient demography, diagnostic criteria, clinical visit details, treatment and relapse parameters. The data quality of the registry was recently validated in 2019.^13^

### Inclusion criteria

Relapsing-remitting multiple sclerosis (RRMS) patients treated with teriflunomide or DMF were included in the analysis. Complete data for all confounder variables used for the derivation of the balancing propensity score was further required. Patients with progressive MS disease at treatment initiation were excluded from the analysis. Where patients recorded starting both teriflunomide and DMF over follow-up, only the first recorded instance of either was included.

### Outcomes and definitions

The study baseline was defined as the start date of the index teriflunomide or DMF. Baseline Expanded Disability Status Scale (EDSS) was defined as the EDSS recorded nearest to the baseline date within 6 months. The primary endpoints of the study were comparative effectiveness and treatment persistence. Comparative effectiveness outcomes included the ARR, time to first on-treatment relapse and 6-month confirmed disability worsening and improvement. Patient-reported outcomes were analysed as secondary outcomes. Six-month confirmed disability progression events were defined as increases of ⩾0.5 points for patients with a baseline EDSS score > 5.5, ⩾1.0 point for those with a baseline EDSS score between 1.0 and 5.5, inclusive, and ⩾1.5 points for those with a baseline EDSS score of 0, confirmed at least 24 weeks subsequent to the visit when the increase was observed. Confirmed improvement was defined as an EDSS decrease of ⩾1.0 confirmed at least 24 weeks following a baseline EDSS score of ⩾2.0. EDSS scores recorded within 30 days after the onset of a relapse were excluded from both the confirmed progression and improvement analyses. Patients were censored at either the date of the outcome event, else the discontinuation date of the index DMT or, where no discontinuation was recorded, the last observed visit. No minimum treatment duration requirement was imposed. Patient-reported outcomes included the Multiple Sclerosis Impact Scale (MSIS-29) physical and psychological scores.^14^

### Power calculation

Drug survival for teriflunomide and DMF in unadjusted data from the Swedish MS Registry has been reported separately.^15,16^ The number of available subjects resulted in a power of 82% with a non-inferiority margin of 5% for comparison of drug survival.

### Statistical analyses

Categorical variables were summarised using frequency and percentage. Continuous variables were summarised using mean and standard deviation (SD), or median and interquartile range (IQR) as appropriate. ARR was compared using the Poisson method. For the primary analysis, confounder imbalance across treatment groups was managed using propensity score matching. The propensity score was derived using a binomial logistic regression where the dependent variable was the index treatment group (teriflunomide or DMF) and the independent variables included known or suspected correlates of the study outcomes selected a priori including age, sex, disease duration, baseline EDSS, pre-index treatment history (the proportion of disease duration on treatment and the class of the drug immediately preceding the index treatment) and pre-baseline relapse activity. The propensity score was then used to match patients on teriflunomide to comparable patients in the DMF cohort on a 1:1 basis. Confounder balance before and after matching was assessed via the derivation of standardised differences.

Treatment persistence, time to first on-treatment relapse, confirmed disability progression and confirmed EDSS improvement were analysed using a marginal Cox model. Hazard proportionality for each outcome was assessed via analysis of scaled Schoenfeld residuals. Kaplan–Meier survival and failure curves were used to visualise time-to-event outcome. Confirmed disability progression and confirmed improvement were further adjusted for visit density where visit density was defined as the count of visits over the follow-up period divided by the follow-up years per patient. As a sensitivity analysis, pairwise censoring of the matched pairs were studied to check for attrition bias, where on-treatment follow-up was censored at the shorter of the two individual treatment follow-up periods for each patient pair. Longitudinal trends in MSIS-29 physical and psychological scores were analysed using generalised estimating equations presuming a linear fit. For all analyses, *p* < 0.05 was considered significant. All analyses were conducted using Stata version 16 (StataCorp, College Station, Texas) and R version 3.6.3 (R Foundation for Statistical Computing, Vienna, Austria).

## Results

### Patients

Of the 358 teriflunomide and 1767 DMF patients eligible for the analysis (Supplementary Table 1), 353 teriflunomide patients were successfully matched to 353 DMF on a 1:1 basis. Standardised differences in the matched sample for all confounder variables used in the derivation of the propensity score were less than 10% (Table 1). There was no difference between the matched teriflunomide and DMF cohorts in terms of age, sex, disease duration, baseline EDSS, pre-baseline treatment history or pre-baseline relapse activity.

**Table 1.** Comparison of baseline characteristics by treatment group in the matched sample.

Characteristic	Category	DMF (*n* = 353)	Teriflunomide (*n* = 353)	Standardised difference
Sex, *n* (%)	Female	243 (68.8)	249 (70.5)	−0.037
	Male	110 (31.2)	104 (29.5)	
Age (years), mean (SD)		47.61 (9.89)	46.71 (10.33)	0.089
Disease duration (years), mean (SD)		11.45 (9.61)	10.86 (8.96)	0.064
EDSS, median (IQR)		1.5 (1, 2.5)	2 (1, 2.5)	−0.034
Proportion of pre-baseline disease duration on treatment, mean (SD)		0.43 (0.35)	0.42 (0.36)	0.026
Pre-index DMT treatment, *n* (%)	Injectables	212 (60.1)	198 (56.1)	−0.054
	Other	33 (9.4)	35 (9.9)	
	Wash–out	29 (8.2)	37 (10.5)	
	Naive	79 (22.4)	83 (23.5)	
Count of relapses in the 12 months prior to baseline, mean (SD)		0.27 (0.55)	0.25 (0.55)	0.026
Count of relapses in the 24 months prior to baseline, mean (SD)		0.31 (0.62)	0.31 (0.61)	−0.000

DMF: dimethyl fumarate; SD: standard deviation; EDSS: Expanded Disability Status Scale; IQR: interquartile range; DMT: disease-modifying treatment.

### Treatment persistence

**Figure 1.**
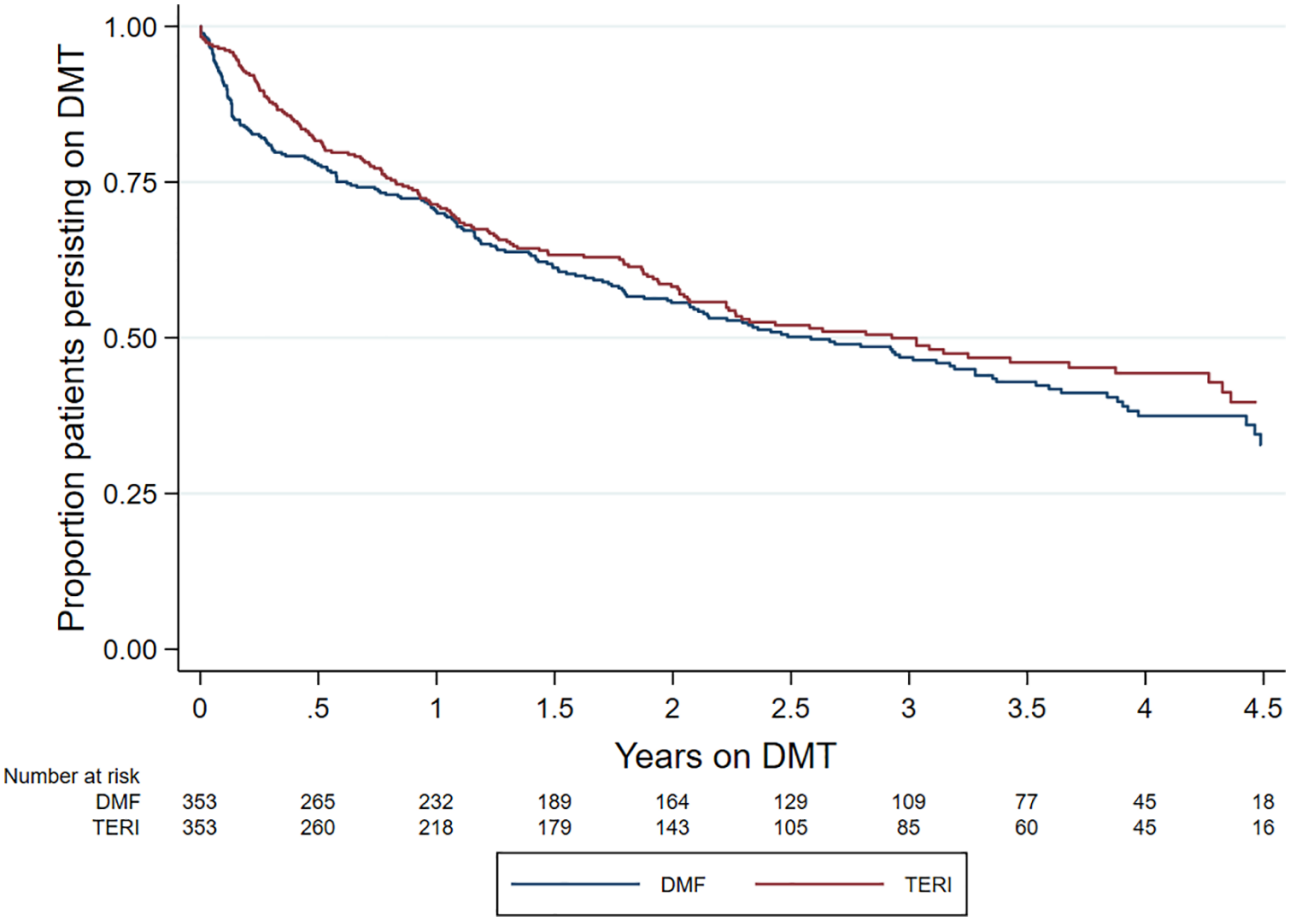
Kaplan–Meier curve – time to treatment discontinuation by drug.

There was no difference in the rate of index treatment discontinuation between the teriflunomide and DMF cohorts (hazard ratio (HR) = 1.12; 95% confidence interval (CI) = 0.91, 1.39; *p* = 0.277; reference = teriflunomide). While the discontinuation rate of DMF (27.44 discontinuations per 100-person years; 95% CI = 23.81–31.64) was marginally higher than the teriflunomide cohort (25.03 per 100 person-years; 95% CI = 21.44–29.23), this did not translate into a significant difference across the observation period (*p*(log-rank) = 0.276) (Figure 1). Within the subset of the patients who discontinued their index treatment, the most frequently reported reason for DMF discontinuation was side effects (89/190; 46.8%) while lack of effectiveness was reported in 39/190 (20.5%) of discontinuations. By comparison, lack of effectiveness was cited as the most frequent discontinuation reason in the matched teriflunomide group (72/160; 45%) followed by side effects (63/160; 39.4%). The third most common reason for discontinuation was ‘other reason’, which was reported in 46/190 (24%) in the dimethyl and 18/160 (11%) in the teriflunomide groups. Planned pregnancy, secondary progressive multiple sclerosis (SPMS) and stable disease were reported as reasons for treatment discontinuation in less than 5% of the matched cohort.

### Relapse

**Figure 2.**
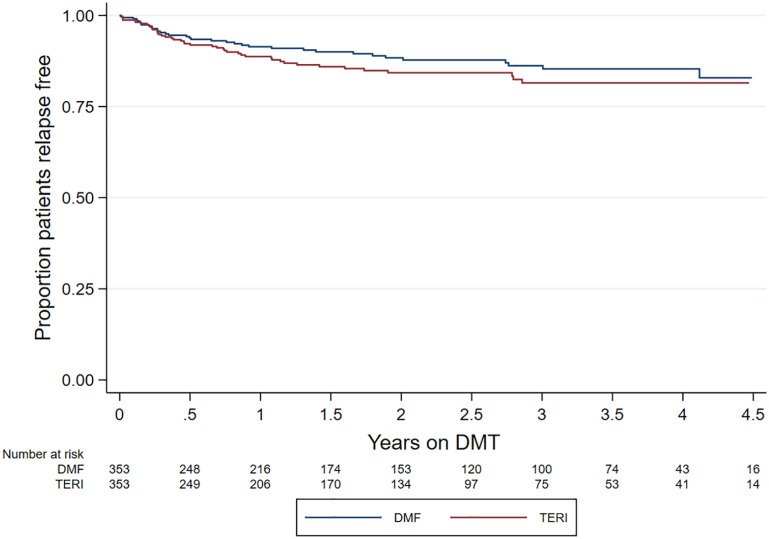
Kaplan–Meier curve – time to first on-treatment relapse by drug.

ARR was comparable (*p* = 0.237) between DMF (0.07; 95% CI = 0.05–0.10) and teriflunomide (0.09; 95% CI = 0.07–0.12) (Table 2). Similarly, there was no difference in time to first on-treatment relapse (HR = 0.78; 95% CI = 0.50–1.21; *p* = 0.270; reference = teriflunomide) (Figure 2).

**Table 2.** Annualised relapse rate (ARR) by treatment group.

Treatment group	Number of on-treatment relapses	Treatment years	ARR (95% CI)	*p*
DMF (*n* = 353)	51	692.33	0.07 (0.05, 0.10)	0.2372
Teriflunomide (*n* = 353)	59	639.21	0.09 (0.07, 0.12)	

CI: confidence interval; DMF: dimethyl fumarate.

### Confirmed disability worsening and improvement

**Figure 3.**
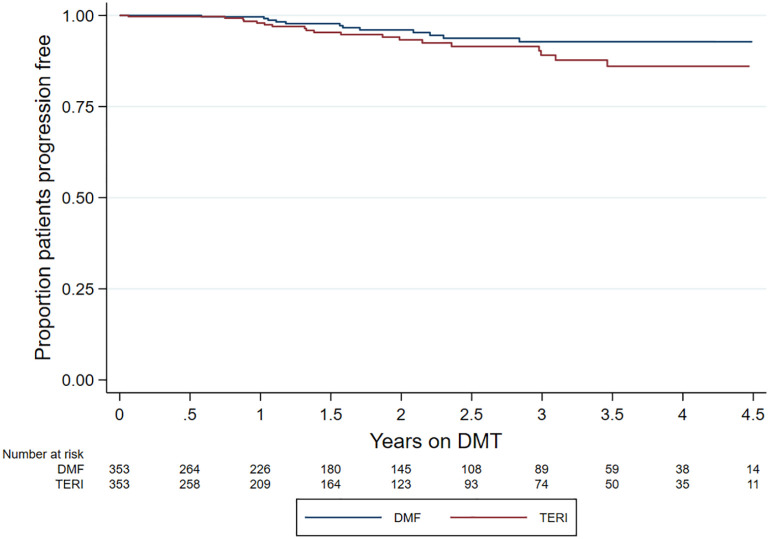
Kaplan–Meier curve – time to confirmed disability worsening by drug.

**Figure 4.**
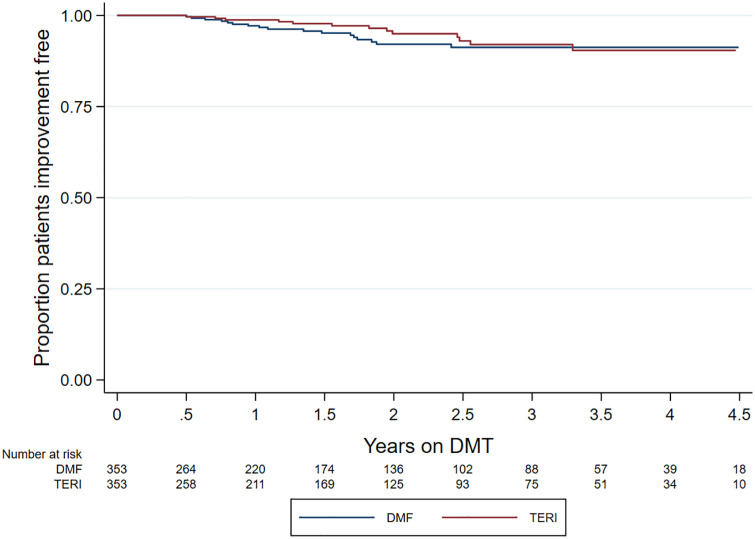
Kaplan–Meier curve – time to confirmed EDSS improvement by drug.

No difference by matched treatment group was observed in the rate of 6-month confirmed disability worsening (HR = 0.55; 95% CI = 0.27–1.12; *p* = 0.100; reference = teriflunomide) (Figure 3). Similarly, there was no difference in 6-month confirmed disability improvement (HR = 1.17; 95% CI = 0.57–2.36; *p* = 0.672; reference = teriflunomide) (Figure 4).

### Patient-reported outcomes – MSIS-29

**Figure 5.**
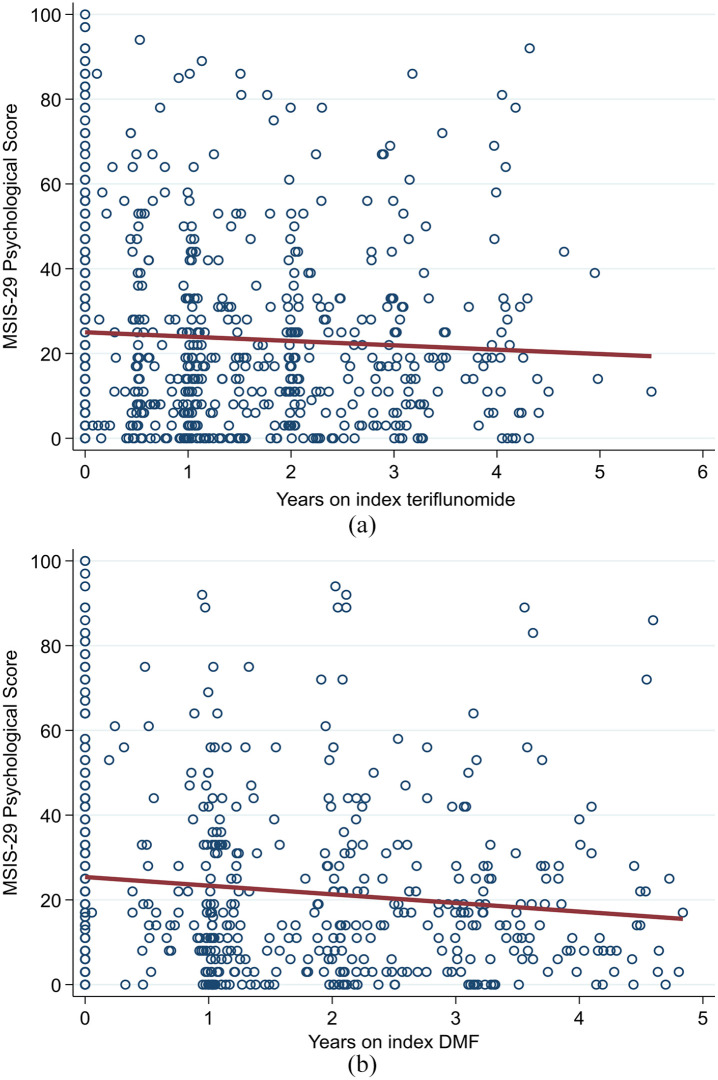
(a) Teriflunomide and (b) DMF – trend in MSIS-29 psychological scores.

**Figure 6.**
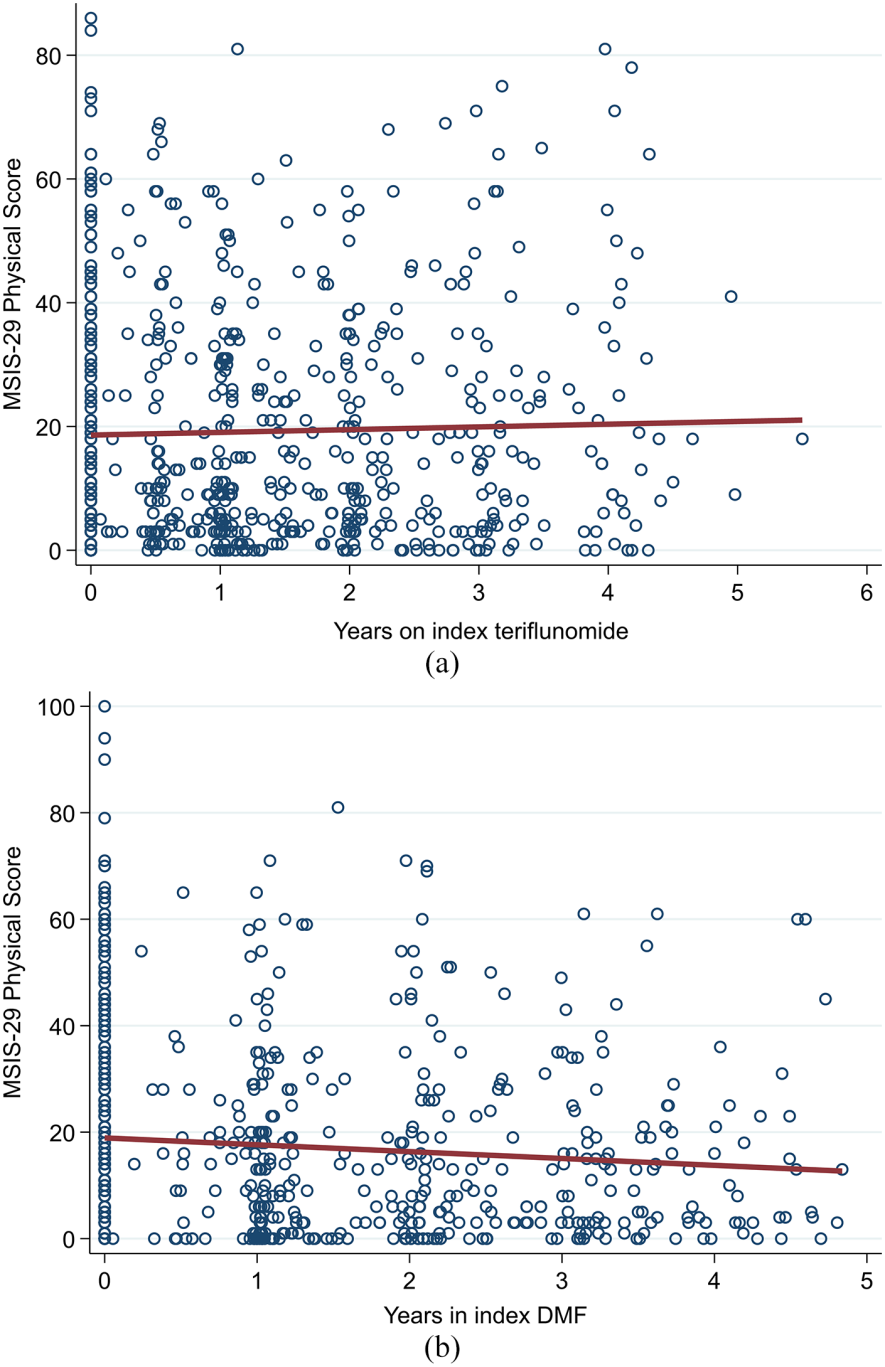
(a) Teriflunomide and (b) DMF – trend in MSIS-29 physical scores.

Both the teriflunomide and DMF cohorts described an overall downwards trend in MSIS-29 psychological scores over the observation period (Figure 5(a) and (b)). There was no significant difference in trend by drug group (β-coefficient = −0.88; 95% CI = −3.16 to 1.40; *p* = 0.450; reference = teriflunomide). By contrast, the teriflunomide cohort described an average upwards trend in MSIS-29 physical scores (Figure 6(a)) compared to a downwards trend in the DMF cohort (Figure 6(b)). However, this difference in trends was not statistically significantly different (β-coefficient = −1.74; 95% CI = −3.68 to 0.19; *p* = 0.077; reference = teriflunomide).

### Sensitivity analysis – pairwise censoring of the matched pair

To test for attrition bias, on-treatment follow-up was censored at the shorter of the two individual follow-up periods for each matched DMF-teriflunomide patient pair (pairwise censoring). Consistent with the primary analysis, no difference was observed between the matched groups in time to treatment discontinuation (HR = 1.01; 95% CI = 0.77–1.32; reference = teriflunomide), first relapse (HR = 0.61; 95% CI = 0.35–1.06), confirmed disability progression (HR = 0.47; 95% CI = 0.19–1.124) or confirmed improvement (HR = 1.83; 95% CI = 0.66–4.96).

## Discussion

Both teriflunomide and DMF have previously demonstrated their efficacy in clinical trials and systematic reviews of RRMS in terms of slowing disability accumulation, decreasing ARRs and reducing the accumulation of MRI lesions and brain volume loss relative to placebo or platform treatments.^2–8,17–20^ What is less known is their efficacy relative to each other in the real-world setting of daily clinical practice, as such direct head-to-head comparisons are limited. This registry-based study observed no difference in any of the key clinical, treatment persistence or patient-reported outcome endpoints studied between the matched teriflunomide and DMF cohorts. While there was a slightly increased rate of premature treatment discontinuation in the DMF group relative to patients treated with teriflunomide (HR = 1.12; 95% CI = 0.91–1.39), this difference was not significant. However, there were some variations in the reported reasons for treatment discontinuation between the treatment groups. Side effects were the most common cause for DMF discontinuation, accounting for almost 50% of all DMF discontinuations. By comparison, lack of effectiveness was the most frequently reported reason for discontinuation in the teriflunomide group (45% of discontinuations), with side effects accounting for 39%. By contrast, only 20% of DMF discontinuations were associated with a lack of effectiveness. However, there was an imbalance in the frequency of reporting ‘other reason’ or not specifying a reason for discontinuation, accounting for 11% and 24% for teriflunomide and DMF discontinuations, respectively. Overall, the distribution of discontinuation reasons between the two treatment groups were largely consistent with Laplaud et al.’s^10^ 2019 cohort study of 1770 patients treated with either teriflunomide or DMF from the French OFSEP registry (which also employed propensity score adjustment to manage confounder imbalance between the treatment groups), which reported a 46% reduction in the odds (odds ratio (OR) = 0.54) of discontinuing treatment due to lack of effectiveness in the DMF cohort relative to teriflunomide. Similarly, Laplaud et al. reported that patients treated with DMF were associated with 1.39 times the odds of discontinuing treatment due to adverse events when compared to teriflunomide-treated patients. The increased frequency of teriflunomide discontinuation observed in our study secondary to lack of treatment effect is also consistent with a recent analysis by Buron et al.^11^ of the Danish Multiple Sclerosis analysis which similarly reported a significantly greater proportion of discontinuations for disease breakthrough in patients treated with teriflunomide (22%; 95% CI = 19.2%–25.0%) compared to DMF (10.2%; 95% CI = 7.6%–12.8%). The observed similarities in overall discontinuation rates are also consistent with a global MSBase comparison of teriflunomide and DMF which also observed no significant difference (*p* = 0.68).^21^

The absence of a difference in clinical relapse, progression and improvement outcomes between the two treatments is also consistent with both the MSBase and OFSEP real-world studies, which similarly observed no difference in either relapse or progression outcomes, despite having larger cohorts available to analyse. This suggests that the absence of such differences in our study is likely not driven by a lack of statistical power. Furthermore, all of these real-world studies were methodologically similar, employing propensity score adjustment to balance the distribution of baseline confounders between the treatment groups. However, the study of the Danish cohort did find a significantly larger ARR in patients treated with teriflunomide (0.16; 95% CI = 0.13–0.20) compared to DMF (0.09; 95% CI = 0.07–0.12) with a much larger sample size (1469 patients on teriflunomide, 767 on DMF). The study by Buron et al. was consistent with the Swedish, French and international MSBase comparisons in reporting no difference in EDSS worsening. The divergence in the Danish data with regard to relapse may be in part a function of the different patterns of usage in Denmark, where teriflunomide has been until recently the recommended first-line treatment in MS. By comparison, patients treated with teriflunomide in Sweden (as well as in France and MSBase) tend to be more heavily pre-treated with other DMT products and are generally older, which may return lower ARRs on second- or subsequent line teriflunomide relative to first-line use.

We were unable to test for differences in MRI lesion count or distribution between the treatment groups due to the lack of sufficient data. Laplaud et al. observed a 40% reduction in the proportion of patients with at least one new T2 hyperintense lesion after 2 years on DMF compared to teriflunomide (odds ratio = 0.60; *p* < 0.001) in the French OFSEP cohort. This may be a partial explanation for the increased rate of discontinuations for lack of effectiveness we observed in the Swedish teriflunomide cohort relative to DMF, although a formal analysis of sufficiently powered MRI data from the Swedish registry would be required to confirm this.

In summary, this population-based real-world study performed on the Swedish MS registry adds to the evidence of similarities in treatment persistence, clinical effectiveness and QoL outcomes of teriflunomide and DMF. Although treatment persistence was comparable between treatments, patients on DMF were more likely to discontinue due to side effects and tolerability issues relative to patients on teriflunomide who were more likely to report lack of effectiveness as the primary driver of treatment interruption.

## Supplemental Material

sj-pdf-1-msj-10.1177_13524585211019649 – Supplemental material for A comparative study of teriflunomide and dimethyl fumarate within the Swedish MS Registry Click here for additional data file. Supplemental material, sj-pdf-1-msj-10.1177_13524585211019649 for A comparative study of teriflunomide and dimethyl fumarate within the Swedish MS Registry by Jan Hillert, Jon A Tsai, Mona Nouhi, Anna Glaser and Tim Spelman in Multiple Sclerosis Journal

sj-pdf-2-msj-10.1177_13524585211019649 – Supplemental material for A comparative study of teriflunomide and dimethyl fumarate within the Swedish MS Registry Click here for additional data file. Supplemental material, sj-pdf-2-msj-10.1177_13524585211019649 for A comparative study of teriflunomide and dimethyl fumarate within the Swedish MS Registry by Jan Hillert, Jon A Tsai, Mona Nouhi, Anna Glaser and Tim Spelman in Multiple Sclerosis Journal

sj-pdf-3-msj-10.1177_13524585211019649 – Supplemental material for A comparative study of teriflunomide and dimethyl fumarate within the Swedish MS Registry Click here for additional data file. Supplemental material, sj-pdf-3-msj-10.1177_13524585211019649 for A comparative study of teriflunomide and dimethyl fumarate within the Swedish MS Registry by Jan Hillert, Jon A Tsai, Mona Nouhi, Anna Glaser and Tim Spelman in Multiple Sclerosis Journal
