# Supplementary figures and images for: A comparative study of teriflunomide and dimethyl fumarate within the Swedish MS Registry
Source: Mult Scler. 2021 Jun 3;28(2):237–46. doi: 10.1177/13524585211019649 (PMC8795225; doi:10.1177/13524585211019649)

## CONSORT DIAGRAM: OBSERVATIONAL STUDY

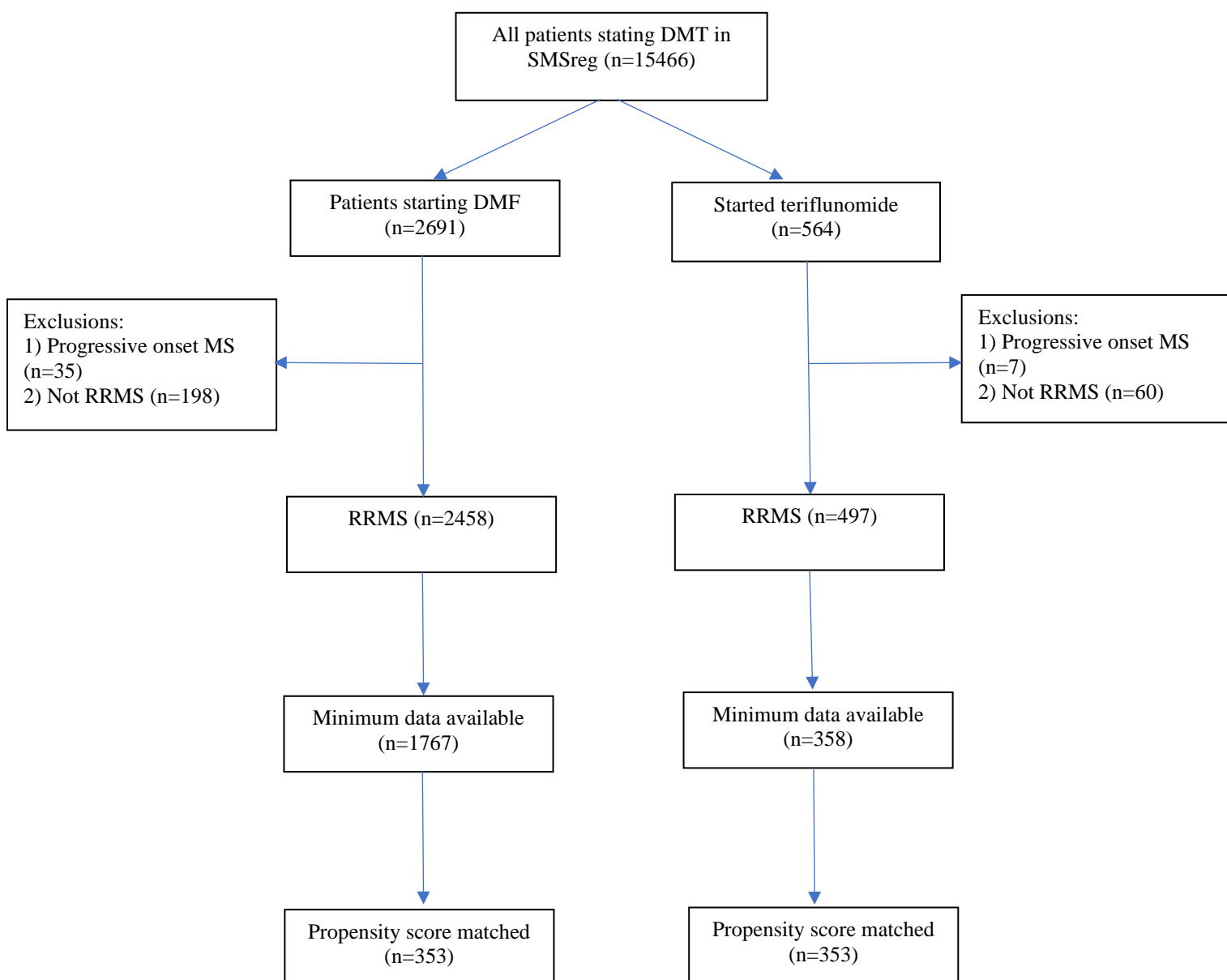

Supplement: sj-pdf-1-msj-10.1177_13524585211019649 – Supplemental material for A comparative study of teriflunomide and dimethyl fumarate within the Swedish MS Registry [file sj-pdf-1-msj-10.1177_13524585211019649.pdf]
