# Supplementary material for: A comparative study of teriflunomide and dimethyl fumarate within the Swedish MS Registry
Source: Mult Scler. 2021 Jun 3;28(2):237–46. doi: 10.1177/13524585211019649 (PMC8795225; doi:10.1177/13524585211019649)
Supplement: sj-pdf-2-msj-10.1177_13524585211019649 – Supplemental material for A comparative study of teriflunomide and dimethyl fumarate within the Swedish MS Registry [file sj-pdf-2-msj-10.1177_13524585211019649.pdf]

Supplementary Table 1: Comparison of baseline characteristics by treatment group in the unmatched sample

Characteristic	Category	DMF (n=1767)	Teriflunomide (n=358)	Standardised Difference
Sex - n (%)	Female	1268 (71.8)	254 (71.0)	0.018
	Male	499 (28.2)	104 (29.1)	
Age (years) - mean (SD)		40.04 (10.93)	47.06 (10.67)	-0.649
Disease duration (years) - mean (SD)		8.08 (8.24)	10.99 (9.07)	-0.336
EDSS - median (IQR)		1.5 (1, 2.5)	2 (1, 2.5)	-0.100
Proportion of pre-baseline disease duration on treatment - mean (SD)		0.36 (0.36)	0.42 (0.36)	-0.180
Pre-index DMT treatment - n (%)	Injectables	837 (47.4)	201 (56.2)	0.243
	Other	162 (9.2)	35 (9.8)	
	Wash-out	143 (8.1)	38 (10.6)	
	Naive	625 (35.4)	84 (23.5)	
Count of relapses in the 12m prior to baseline - mean (SD)		0.38 (0.63)	0.25 (0.55)	0.225
Count of relapses in the 24m prior to baseline - mean (SD)		0.52 (0.77)	0.31 (0.60)	0.300
