# Supplementary material for: A comparative study of teriflunomide and dimethyl fumarate within the Swedish MS Registry
Source: Mult Scler. 2021 Jun 3;28(2):237–46. doi: 10.1177/13524585211019649 (PMC8795225; doi:10.1177/13524585211019649)
Supplement: sj-pdf-3-msj-10.1177_13524585211019649 – Supplemental material for A comparative study of teriflunomide and dimethyl fumarate within the Swedish MS Registry [file sj-pdf-3-msj-10.1177_13524585211019649.pdf]

Supplementary Table 2: Logit model for derivation of propensity score

**Explanatory covariate**	**log-odds (95% CI) p-value**
Age (years)	-0.06 (-0.07, -0.04) <0.001
Disease duration (years)	0.00 (-0.01, 0.02) 0.854
Female sex	0.08 (-0.18, 0.34) 0.563
Baseline EDSS	0.07 (-0.01, 0.15) 0.108
Proportion of pre-baseline disease duration on treatment	0.02 (-0.42, 0.45) 0.944
Pre-index DMT treatment	0.08 (-0.04, 0.21) 0.186
Count of relapses in the 12m prior to baseline	-0.38 (-0.89, 0.12) 0.139
Count of relapses in the 24m prior to baseline	0.57 (0.13, 1.00) 0.011
